# A Nomogram for the Rapid Prediction of Hematocrit Following Blood Loss and Fluid Shifts in Neonates, Infants, and Adults

**DOI:** 10.7759/cureus.7780

**Published:** 2020-04-22

**Authors:** Francesco M Egro, Elizabeth M Kenny, Ernest C Manders, Ernest Manders

**Affiliations:** 1 Plastic Surgery, University of Pittsburgh, Pittsburgh, USA; 2 Otolaryngology, The Christ Hospital, Cincinnati, USA

**Keywords:** critical care medicine, nomograms, hematocrit, fluid shifts, blood volume

## Abstract

Introduction

There is often a need for a simple means of predicting hematocrit (Hct) following blood loss, administration of intravenous fluids, or fluid shifts. The aim of this study is to introduce a nomogram for the rapid prediction of blood volume and packed red cell volume appropriate for a given patient's body weight and Hct in both the pediatric and adult populations.

Methods

A nomogram for prediction of Hct was created using the following variables: 1) blood volume determined from bodyweight, 2) estimated blood loss, and 3) initial Hct.

Results

Hct was calculated after blood loss, administration of intravenous fluids, or fluid shifts using the pediatric and adult nomograms. Alternatively, the nomograms can be used to back-calculate blood or fluid loss if Hct is known. The nomogram allows for adjustment for measured and insensible fluid losses and fluid administration.

Conclusions

The nomogram helps to predict the Hct and fluid requirements in neonates, children, and adults with blood loss, fluid administration, and rehydration following dehydration. It allows for the calculation of Hct after fluid shifts in a simple, fast, and portable manner. We believe it can be a useful adjunct to monitor the fluid balance in all patients, especially in resource-limited settings where laboratory equipment may not be available.

## Introduction

Hemorrhage and dehydration are unfortunate yet common presentations in many patients. While developed countries have easy access to fluid resuscitation and tools to monitor the response, developing countries often lack the equipment to run common blood levels and, hence, the determination of adequate response to resuscitation becomes far more challenging.

Hematocrit (Hct) represents the volume of red blood cells (RBCs) in the blood and is therefore reported as a percentage. Hct is considered one of the most precise methods of determining the degree of polycythemia or anemia [1]. Optimum Hct values differ with gender, with normal ranges spanning from 46.0 ±4.0% for adult males to 40.0 ±4.0% for adult females (mean ±2 SD) [2]. Low hematocrit and hemoglobin levels usually indicate a decreased production, excessive loss, or destruction of RBCs. Thus, detecting changes in the Hct is important not only for diagnostic purposes but also for therapeutic purposes including estimation of transfusion or resuscitation needs [3].

From the emergency room to the operating room to the intensive care unit, there is a need for a simple means of calculating a patient's Hct. Hct was historically measured directly, but indirect measurements have grown in popularity as modern lab equipment has become readily available. Direct measurement of Hct involves microhematocrit centrifugation, where the blood sample is spun at high speed in a heparinized capillary tube to allow for the sedimentation of the sample. The proportion of RBCs in the blood can then be calculated by dividing the length of the RBC column by the length of the whole blood column, and Hct as a percentage can be calculated by multiplying this value by 100 [4]. Indirect measurement utilizes an automated hematology analyzer to calculate Hct using the following equation: Hct (%) = (RBC count x mean RBC volume) / (total sample volume) [5]. While direct and indirect Hct measurements are highly accurate, the time-consuming and costly nature of laboratory testing can limit their clinical utility, particularly in resource-limited settings where laboratory equipment may not be available.

A nomogram will fill the need for a simple, rapid, and cost-effective means of predicting Hct measurements. A nomogram offers a ready means of imputing data for graphical calculation. The use of a nomogram is faster than using a handheld calculator or computer. A nomogram is portable as well and can be readily available wherever needed.

The aim of this study is to introduce a nomogram that will allow physicians and nurses to use the estimated blood loss to calculate the patient's Hct following fluid resuscitation, as well as following fluid shifts (e.g., intravenous fluid administration, dehydration, or gastrointestinal losses).

## Materials and methods

The calculation of Hct following blood loss and fluid shifts requires knowledge of the blood volume (BV) appropriate for the patient. The patient's weight in kilograms (kg) or pounds (lb) is also required.

It is well known that blood volume varies with age [6]. A premature infant may have a BV of 85-100 ml/kg [7,8]. By one month of age after full-term delivery, however, the BV is usually 75 ml/kg [9]. This value persists for most of the lifespan of the individual, regardless of gender. In the elderly, especially females with a higher percentage of body fat, the BV may fall to 70 ml/kg. The value of 75 ml/kg has been used for the construction of the nomogram here. The nomogram will, therefore, underestimate the BV of the premature infant soon after its birth. As a consequence, the fluid replacement for the dehydrated neonate may be slightly underestimated, and for the obese female, it may be slightly overestimated. These errors are negligible, however, and the values obtained from the nomograms should prove to be valuable approximations, even in the case of the premature neonate and those with high adiposity.

Calculation of blood volume

Assuming a BV of 75 ml/kg, the graphical determination is simple, requiring only that the user of the nomogram reads the BV opposite the body weight.

Derivation of formulas

We begin by examining the change in Hct occurring with blood loss and replacement of crystalloid or plasma. RBC volume denotes red blood cell volume in milliliters and BV denotes whole blood volume in milliliters. The Hct is defined as:

$Hct=\frac{RBC volume, ml}{BV, ml}\times 100$

Assuming restoration of blood volume, the final Hct (Hctf) is calculated, where the subscript i denotes initial RBC volume and the subscript f denotes Hctf after blood loss:

$Hct_{f} =\frac{{(RBC volume)_{i}-(RBC volume)_{lost}}}{BV}\times 100$

The RBC volume lost = (Hcti) (BVlost). Therefore:

$Hct_{f} =\frac{{(Hct_{i})(BV_{i})-(Hct_{i})(BV_{lost})}}{BV_{i}}\times 100$

In summary:

$Hct_{f} =\frac{{Hct_{i}(BV_{i}-BV_{lost})}}{BV_{i}}\times 100$

## Results

Use of the nomogram after blood loss and equilibration

**Figure 1:**
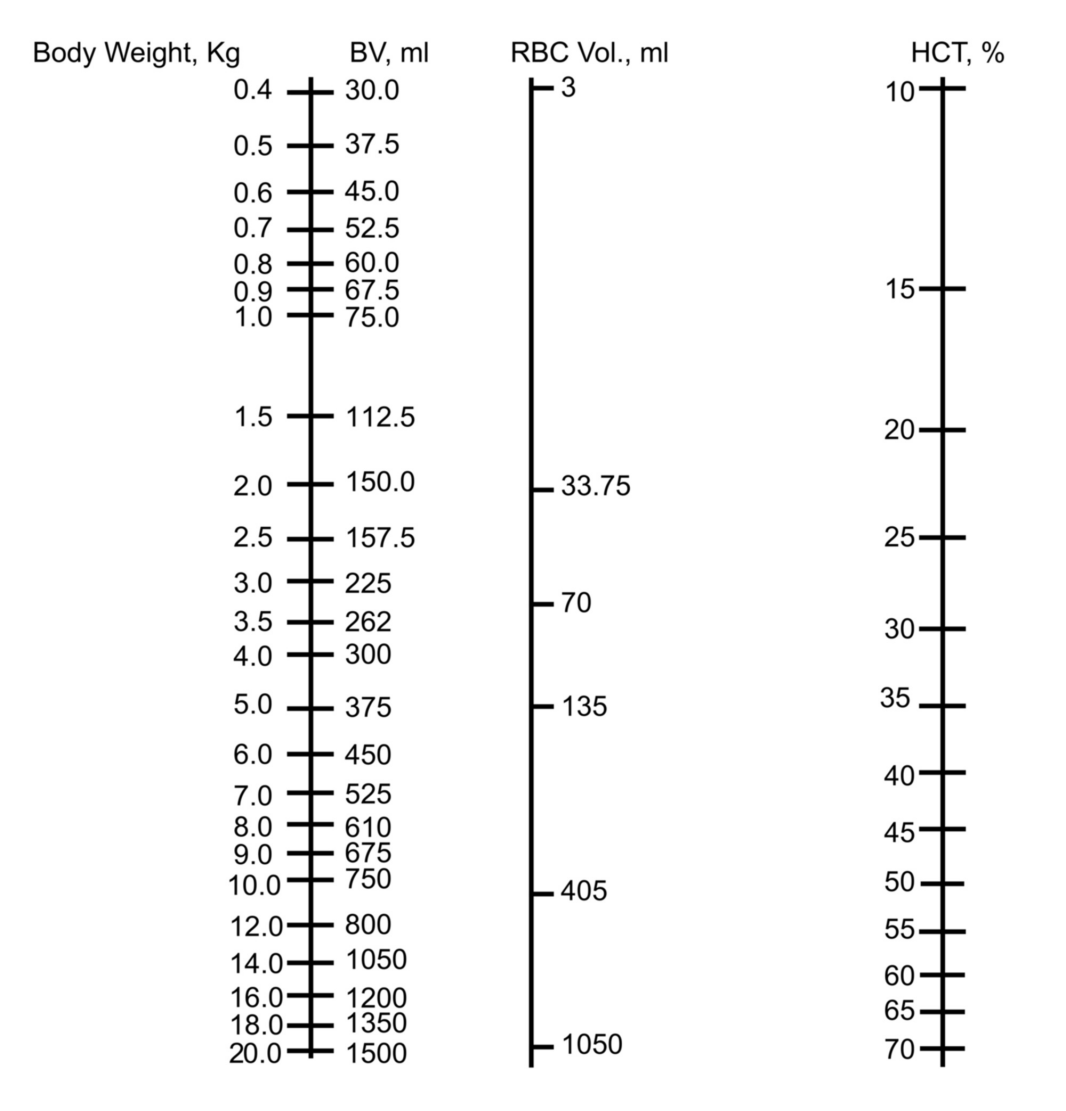
The nomogram for determining blood volume and hematocrit in neonates and infants BV: blood volume; RBC: red blood cells; Hct: hematocrit

**Figure 2:**
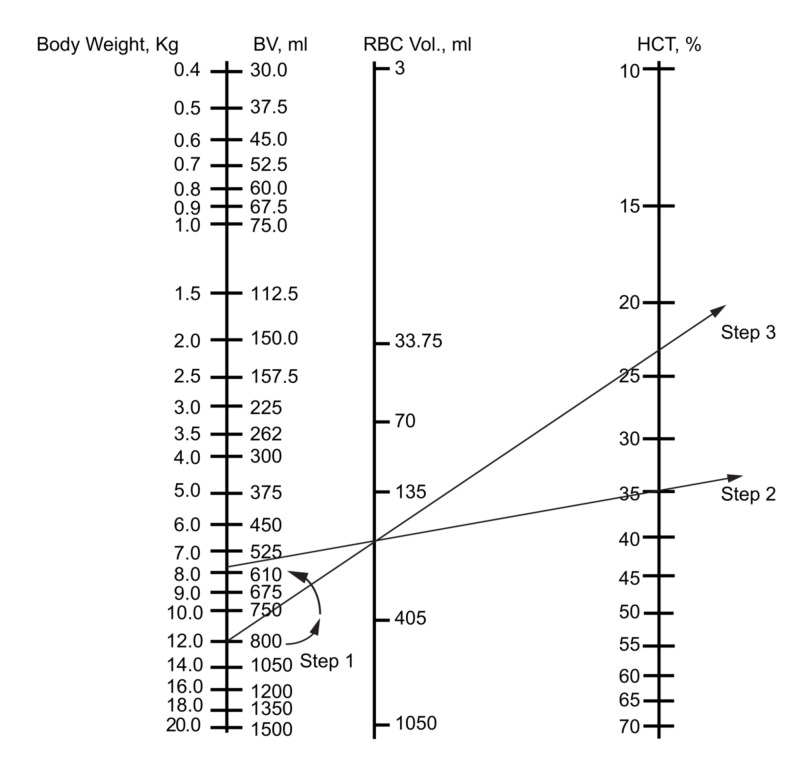
Use of the nomogram to predict the hematocrit following blood loss, assuming fluid replacement and/or recruitment Clinical scenario: what is the Hct after equilibration for a 12-kg infant who has lost 200 ml of blood with an initial Hct of 35%? Step 1: subtract the blood loss in ml from the BV Step 2: connect the current BV and the initial Hct on the nomogram using a straight edge Step 3: pivot the straight edge against a pencil tip on the RBC volume line, connecting the original weight and the new Hct appearing as a consequence of the smaller RBC mass BV: blood volume; RBC: red blood cells; Hct: hematocrit

To use the nomogram for neonates and infants to predict Hct after blood loss and fluid recruitment, we have to determine the normal BV opposite the body weight first (Figure 1). We then subtract the blood loss from the BV scale (Figure 2) (step 1). Place the ruler on the scale at a point denoting the corrected blood volume. The remaining RBC volume is determined by connecting the corrected BV to the original hematocrit (Hcti) (Figure 2) (step 2). The straight edge crosses the intermediate line at a point indicating the red cell volume. Having subtracted the estimated blood loss from the BV scale, the RBC volume will be corrected to a lower volume. We place the point of the pencil on the point where the straight edge crosses the RBC volume line. The ruler is now pivoted on the pencil point and the left-hand end is brought over the original BV (or body weight in kg). The right-hand end of the ruler now lies on the Hctf (Figure 2) (step 3). It should be noted that this simple scheme assumes that the BV has been restored with the recruitment of interstitial fluid or intravenous plasma or crystalloid, not whole blood or packed cells.

In the event of unmeasured blood loss, the amount lost can be estimated by measurement of the current Hct and back-calculation with the nomogram. For this determination, we join the present Hct with body weight. The present post-loss RBC volume is noted. We then move the straight edge down to an assumed or actual pre-loss Hct. The pre-loss RBC volume is noted. By dividing the difference in RBC volume (pre-loss RBC volume minus post-loss RBC volume) by the pre-loss Hct (taken as a decimal, not a percentage), we estimate the BV actually lost.

Use of the nomogram after fluid administration without blood loss

**Figure 3:**
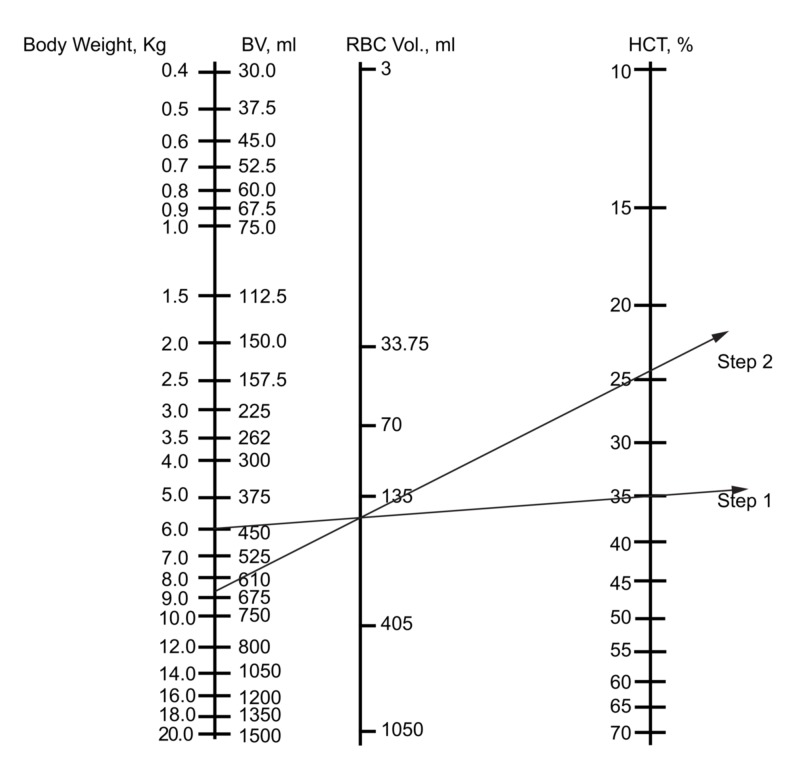
Use of the nomogram to predict the hematocrit in the setting of fluid administration without blood loss Clinical scenario: what is the Hct after 200 ml of fluid administration to a 6-kg infant with no blood loss and an initial Hct of 35%? Step 1: lay the ruler from the body weight to the initial Hct Step 2: rotate the straight edge about the pencil tip on the RBC volume line so that the edge crosses the BV line at the number denoting the sum of the original BV and the fluid infused. The right end of the ruler indicates the resultant Hct BV: blood volume; RBC: red blood cells; Hct: hematocrit

Not infrequently, a patient may receive a substantial intravenous infusion while having little or no blood loss or insensible loss. This is not unusual during general anesthesia when the capacitance of the vascular system increases dramatically [10]. The Hct will be low in the recovery room, but not because of blood loss. The use of the nomogram will help prevent needless worry and unnecessary transfusion. Figure 3 (step 1), shows the first step in utilizing the nomogram to predict Hct for patients undergoing intravenous fluid administration without blood loss. The straight edge is passed from the body weight in kg to the Hcti. The pencil tip is used to mark the RBC volume. In Figure 3 (step 2), the ruler or straight edge is rotated about the pencil point or hatch mark on the RBC volume line to the point denoting the original BV plus the amount of fluid infused on the left. The right end of the straight edge indicates the Hctf.

Use of the nomogram in the operating room with blood loss and fluid administration

**Figure 4:**
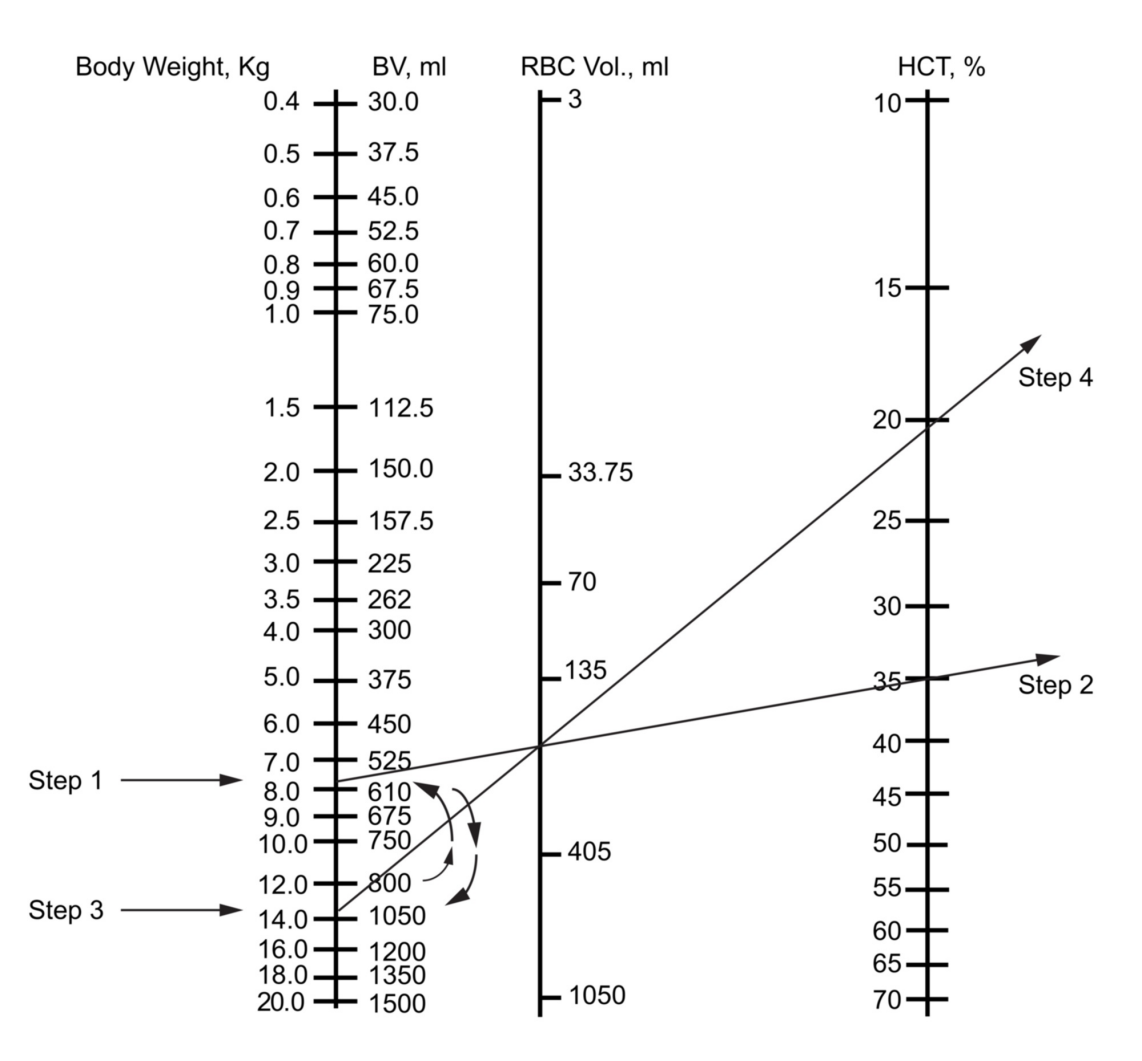
Use of the nomogram to predict the hematocrit in the operating room with both blood loss and fluid administration Clinical scenario: what is the Hct in the case of a 12-kg infant with initial Hct of 35% following 200 ml of blood loss and 400 ml of crystalloid administration? Step 1: the loss of 200 ml of blood is noted by subtraction from the initial BV Step 2: determination of RBC volume after blood loss with a line from resultant BV to the initial Hct. Place a pencil point on the intersection with the RBC volume line Step 3: the administration of 400 ml of crystalloid is added to the blood volume Step 4: rotate the straight edge to the new blood volume, noting the final Hct where the straight edge crosses the Hct line BV: blood volume; RBC: red blood cells; Hct: hematocrit

In the operating room, there are often both blood losses and simultaneous fluid administrations. The nomogram presented here allows for the calculation of the expected Hctf, based on the estimated blood loss and other fluid balance figures such as urinary loss and intravenous crystalloid administration. First, we adjust for blood loss by subtracting the loss from the patient's BV opposite the body weight in kg (Figure 4 (step 1). Then we determine the new RBC volume by laying the straight edge from the new BV to the Hcti (Figure 4) (step 2). The resulting RBC volume is noted and marked.

Net fluid is added to the blood volume (Figure 4) (step 3). Here note should be made of approximations inherent in the foregoing sentence. Large volumes of urine lost and large insensible losses from evaporation or third spacing may be subtracted from the total volume of fluid administered. This step requires an experienced estimation of insensible losses due to the evaporation and fluid transudation into the interstitial compartment [11]. The estimates will depend on such factors as the operation performed, the length of the procedure, and the size of the patient. Once the new BV is estimated, the left-hand end of the straight edge is moved to the volume. The ruler is pivoted on the RBC volume and the new Hct is noted (Figure 4) (step 4).

If whole blood is transfused, this should be added to the BV (or subtracted from the blood loss before this figure is subtracted from the predicted BV as in Figure 2, step 1). If packed RBCs are transfused, their volume may be calculated using the following formula given the fact that the Hct of packed RBCs is approximately 65% [12].

$(packed RBCs, ml)(0.65)=RBC volume, ml$

The RBC volume transfused may be added to the RBC volume determined after subtraction of whole blood losses and before accounting for fluid administration and determination of the Hctf.

Use of the nomogram in the case of fluid loss

**Figure 5:**
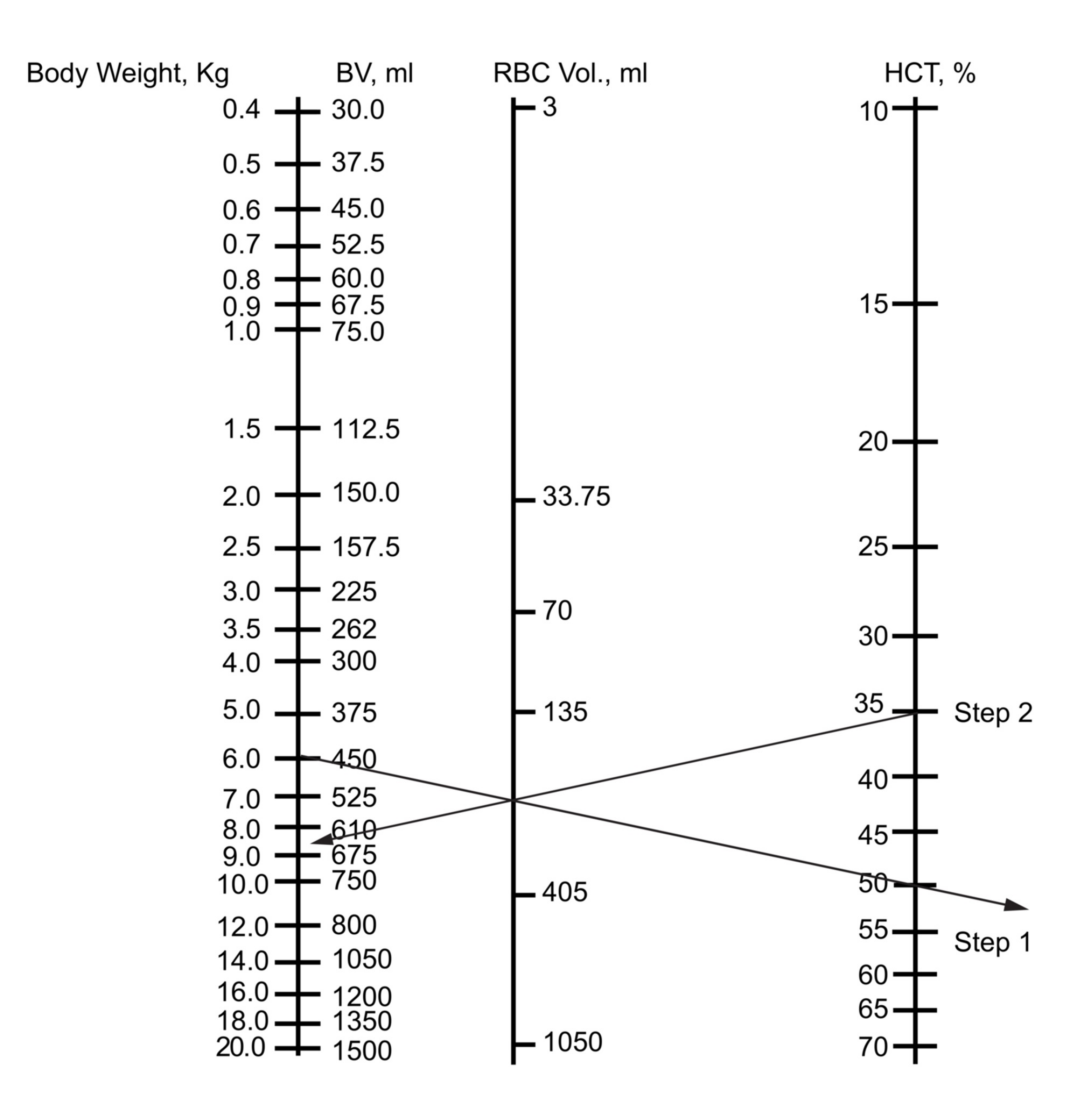
Use of the nomogram to calculate fluid loss Clinical scenario: what is the total fluid loss in a dehydrated infant with a weight of 6 kg and Hct of 50% at presentation? Step 1: draw a line from the current weight to the current Hct to determine the RBC volume Step 2: the right-hand end of the straight edge is moved upward to the Hct judged to be likely present before dehydration, pivoting about the point marking the RBC volume. The anticipated weight on rehydration is indicated on the left-most scale BV: blood volume; RBC: red blood cells; Hct: hematocrit

The nomogram allows for the calculation of the Hct expected from fluid shifts, such as loss of total body water from urinary and gastrointestinal losses. To make these calculations, we first enter the weight (kg) on the left scale and place the right-hand end of the ruler or straight edge over the measured Hct. The RBC volume is marked (Figure 5) (step 1). The right-hand end is moved upward to the Hct judged present for the patient before the losses of concern occurred (Figure 5) (step 2). The left end of the ruler gives a new body weight and BV based on the RBC volume. Subtraction of the preceding body weight from the calculated weight yields a difference, which may be ascribed to fluid loss. A 2-kg difference, therefore, indicates a 2-liter deficit of total body water.

Use of the nomogram in adults

**Figure 6:**
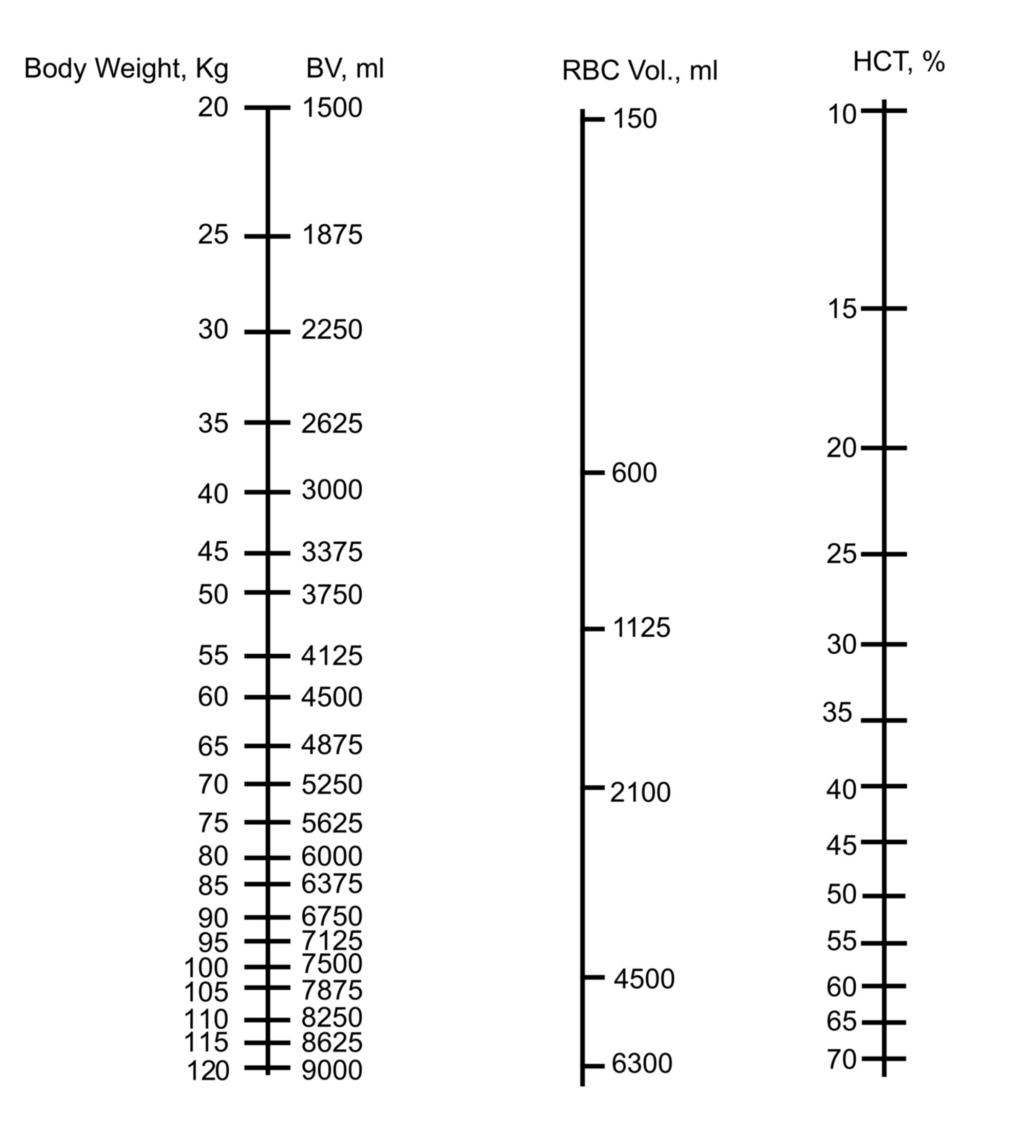
The nomogram for determining blood volume and hematocrit in adults BV: blood volume; RBC: red blood cells; Hct: hematocrit

Because of the utility of the nomogram for prediction of Hct in neonates and infants, we created a similar nomogram for use in the adult population (Figure 6). The adult nomogram can be used in the previously presented fluid-balance scenarios: blood loss, fluid administration, combined blood loss and fluid administration, and fluid loss.

## Discussion

The nomogram presented here allows for the rapid calculation of the expected Hct based on the size of the patient and changes in intravascular volume from blood loss, other fluid losses, and administration of intravenous fluids. A knowledge or estimate of the Hcti is required. The neonate and infant nomogram allows for more precision in the display of the numerical values for small patients in this age group. Furthermore, we were able to adapt the nomogram for use in the adult population as well.

The nomogram may be used for calculating the eventual Hctf after blood loss when the final equilibrium has occurred from fluid administration, recruitment from the interstitium, and oral intake and absorption. While the method is accurate, its precision depends upon equilibration and restoration of blood volume by one or more of the means above. The nomogram may also be useful for calculating the Hctf after fluid shifts. In the case of diabetes insipidus or in a multiply injured trauma patient, the use of the nomogram may help predict Hctf on the basis of fluid balance alone.

Alternatively, if Hcti is known or estimated and the current Hctf is known, then one may work backward as it were to calculate fluid loss or gain as illustrated in Figure 3. This use of the nomogram will aid in fluid management of patients with impaired renal function and decreased urine output. The degree of dehydration and the amount of crystalloid necessary for fluid resuscitation of the dehydrated patient can also be estimated from the nomogram. It should be noted that the physician will still need to select a fluid appropriate for the electrolyte status of a given patient.

While on a mission trip, the senior author encountered the challenge of monitoring fluid shifts without access to hematologic tests. This nomogram was developed to address the need to monitor resuscitation of severely dehydrated neonates in a rural, low-resource, and underdeveloped area where laboratory testing was unavailable. This paper demonstrates how in a few simple steps one can monitor the fluid balance in an easy, fast, and inexpensive manner. Although the ideation of the nomogram targets underserved areas of the world where there is a lack of medical equipment, the nomograms can easily be applied in developed countries as well, especially in the emergency room, operating room, and intensive care settings. While this nomogram was created with the pediatric population in mind, the concepts are valid in the adult population as well. To this end, we created a nomogram to aid in the monitoring of fluid balance in adults.

Despite their easy, fast, and inexpensive application, the presented nomograms are not without limitations. First, the nomogram assumes that BV is constant (75 ml/kg). As previously mentioned, this can underestimate the BV in premature infants where BV is higher than term infants, children, and adults [13,14]. Next, the nomogram considers BV as a sole factor of body weight; however, estimations of BV using both weight and height (or surface area) may better reflect the true BV than weight alone, in both adults and children [15,16]. Even when both weight and height are taken into account in the calculation of BV, studies have shown that additional factors related to body habitus such as relative muscle and adipose tissue may affect the BV [17]. The confounding factor of body habitus is likely less relevant in the neonate and infant population, but it may be important in adults where extremes of body habitus are more common. Future research will target the validation and evaluation of the efficacy of the nomogram in the clinical context.

## Conclusions

To conclude, the nomogram helps to predict the Hct and fluid requirements in neonates, children, and adults with blood loss, fluid administration, and rehydration following dehydration. It allows for the calculation of Hct after fluid shifts in a simple, fast, and portable manner, and it can be a useful learning tool for trainees to help understand the interplay between Hct and fluid status in pediatric and adult patients undergoing fluid resuscitation. We believe it can be a useful adjunct to monitor the fluid balance in all patients, especially in resource-limited settings where laboratory equipment may not be available.
